# Major Cys protease activities are not essential for senescence in individually darkened Arabidopsis leaves

**DOI:** 10.1186/s12870-016-0955-5

**Published:** 2017-01-06

**Authors:** Adriana Pružinská, Takayuki Shindo, Sherry Niessen, Farnusch Kaschani, Réka Tóth, A. Harvey Millar, Renier A. L. van der Hoorn

**Affiliations:** 1 The Plant Chemetics laboratory, Max Planck Institute for Plant Breeding Research, 50829 Cologne, Germany; 2 The Skaggs Institute for Chemical Biology and Department of Chemical Physiology, The Center for Physiological Proteomics, The Scripps Research Institute, La Jolla, 92037 California USA; 3 The Australian Research Council Centre of Excellence in Plant Energy Biology, The University of Western Australia, Perth, WA Australia; 4 The Plant Chemetics Laboratory, Department of Plant Sciences, University of Oxford, OX1 3RB Oxford, UK; 5 Department of Plant Developmental Biology, Max Planck Institute for Plant Breeding Research, 50829 Cologne, Germany

**Keywords:** Senescence, Activity-based protein profiling, Papain-like proteases, Vacuolar processing enzymes

## Abstract

**Background:**

Papain-like Cys Proteases (PLCPs) and Vacuolar Processing Enzymes (VPEs) are amongst the most highly expressed proteases during leaf senescence in Arabidopsis. Using activity-based protein profiling (ABPP), a method that enables detection of active enzymes within a complex sample using chemical probes, the activities of PLCPs and VPEs were investigated in individually darkened leaves of Arabidopsis, and their role in senescence was tested in null mutants.

**Results:**

ABPP and mass spectrometry revealed an increased activity of several PLCPs, particularly RD21A and AALP. By contrast, despite increased *VPE* transcript levels, active VPE decreased in individually darkened leaves. Eight protease knock-out lines and two protease over expressing lines were subjected to senescence phenotype analysis to determine the importance of individual protease activities to senescence. Unexpectedly, despite the absence of dominating PLCP activities in these plants, the rubisco and chlorophyll decline in individually darkened leaves and the onset of whole plant senescence were unaltered. However, a significant delay in progression of whole plant senescence was observed in *aalp-1* and *rd21A-1/aalp-1* mutants, visible in the reduced number of senescent leaves.

**Conclusions:**

Major Cys protease activities are not essential for dark-induced and developmental senescence and only a knock out line lacking AALP shows a slight but significant delay in plant senescence.

**Electronic supplementary material:**

The online version of this article (doi:10.1186/s12870-016-0955-5) contains supplementary material, which is available to authorized users.

## Background

Senescence is the final stage in the development of cells, tissues, and organs and in the case of monocarpic species, entire plants. Leaf senescence is characterized by extensive protein degradation that enables remobilisation of nutrients, especially nitrogen, for use in other parts of the plant, such as newly developing organs, seeds or storage tissues. Protein degradation during senescence involves the disassembly and degradation of the photosystem and metabolic pathways and all other proteins of the living cell until no proteins remain for recycling [1]. Four pathways of chloroplast breakdown have been identified in Arabidopsis. These pathways involve autophagy, senescence-associated vacuoles (SAVs), chloroplast vesiculation, and selective chloroplast destruction via a 13-lipoxygenase [2–5]. However, despite the importance of this process, the proteases responsible have not all been identified and characterized.

Gene expression studies indicated that Cys proteases are amongst the most abundant proteases during leaf senescence [6]. Senescence-associated Cys proteases are papain-like Cys proteases (PLCPs, protease family C1A in MEROPS Database [7]), legumains or vacuolar processing enzymes (VPEs, family C12), metacaspases (family C14), calpains (family C2) and proteases related to ubiquitin-dependent pathways (families C13, C19 and C85) [6, 8–15]. Reduced protein degradation in senescing leaf segments of wheat can be achieved upon treatment with Cys protease inhibitors, indicating the involvement of Cys proteases in senescence [16].

PLCPs are the major enzymes associated with bulk protein degradation during senescence [14]. However, PLCPs are involved also in other physiological processes such as germination and plant defence [17]. Arabidopsis *AALP (SAG2)* and *SAG12*, both encoding PLCPs, are used as standard markers of leaf senescence [18–20]. *SAG12* is exclusively expressed in senescent leaves and the encoded protein is localized to senescence-associated vacuoles (SAVs) [2]. By contrast, *AALP* (*SAG2*) transcription occurs in leaves at all developmental stages but increases during natural and stress related senescence in (reviewed in [8]). In sweet potato, increased expression of two PLCPs, *SPCP2* and *SPCP3* (homologs of *RD19* and *RD21A*, respectively) occurs in both natural and stress-induced senescing tissues [21, 22] similarly to a soybean PLCP called *GMCP3* [23]. Additionally, four putative cDNAs encoding PLCPs (*BoCP1, BoCP2, BoCP3, BoCP4*) have been isolated from senescing broccoli floret tissue that are similar to Arabidopsis RD19 and RD21A [24]. Arabidopsis RD21A was found in the vacuoles of senescing leaves and is synthesized as a 57-kDa precursor that is slowly processed into a 33-kDa mature protein (mRD21A) via a 38-kDa intermediate (iRD21A) [25]. These intermediates accumulate in the vacuole as aggregates, however during leaf senescence they are released as a soluble protease upon removal of the granulin domain [25]. In a similar manner, SoCP is a 41-kDa protease with a granulin domain that is transcriptionally induced in senescent leaves of *Spinacia oleraceae* [26].

In Arabidopsis, VPEs mediate processing of vacuole-localised proteins during seed germination and developmental or pathogen-mediated programmed cell death [27–30]. It has been proposed that *γ*VPE might also activate proteases involved in protein recycling during senescence [27]. Transcript levels of *γVPE* was increased in leaves during development of Arabidopsis [31]. Moreover, *γVPE* is highly induced in petals of tobacco as they progress in development and it was suggested using it as a senescence marker for petal senescence [32].

Protease activity is regulated by transcriptional and translational processes, but also by post-translational modifications and by protease inhibitors [33]. PLCPs are synthesized with an autoinhibitory prodomain that must be proteolytically removed to activate the enzymes [34]. Senescence-related PLCPs with granulin domain in complex with cystatin have been purified from leaves of spinach and this protease was activated by releasing cystatin from the complex [35]. Similarly, the role of cystatins in modulating of cysteine protease activity during senescence is proposed in barley [36]. Overexpression of rice cystatin in tobacco inhibits Cys protease activity, delaying the decline of Rubisco and two Rubisco activase proteins [37]. AtSerpin1 interacts with RD21A and it is expected that other serpins might regulate senescence [38].

Because of this post-translational regulation, accumulation of proteases or protease-encoding transcripts does not necessarily correlate with protease activity. To study protease activities, rather than transcript or protein accumulation, we applied activity-based protease profiling (ABPP). ABPP is based on the use of fluorescent or biotinylated chemical probes that react irreversibly with the active site of enzymes in a mechanism-dependent manner [39–41]. Here, we applied ABPP to study protease activities during leaf senescence induced by individually darkening leaves of Arabidopsis and we used PLCP and VPE mutants and over expressing lines to confirm the origin of these signals and determine the relative contribution of these proteases to leaf senescence.

## Methods

### Plant material and growth conditions

All *Arabidopsis thaliana* transgenic and knockout lines were Columbia ecotype and are summarized in Additional file 1: Table S2. The *rd21-1, aalp-1, sag12-1*, and *ctb3-1* mutants [42]; the *rd21-1/aalp-1* double mutant [43]; the γVPE overexpressor (*35S::γVPE*, [44]), the VPE quadruple knockout (*qvpe*) mutant lacking all four VPEs [45], and the triple mutant *ctb1/2/3* (line #65-4, [46]) have been described previously. The 35S::RD21 overexpressor line was generated by transforming Col-0 with pRH628 [43] using the flowerdip method. Transgenic plants were selected on kanamycin and homozygous lines were characterized by ABPP (Additional file 2: Figure S3). Plants were grown for six or eight weeks in controlled growth cabinets. Three sets of growth conditions were used: 12/12 hours day/night cycle at 24 °C/20 °C temperatures, 16/8 hours day/night cycle at 22 °C/18 °C hours (long day), and 8/16 day/night cycle at 22 °C/18 °C (short day).

### Chlorophyll quantification

A Soil Plant Analysis Development (SPAD) meter (502 Plus Chlorophyll Meter, Spectrum Technologies) was used to determine the relative chlorophyll content [47]. The SPAD analyser measures leaf transmission at two wavelengths (650 and 940 nm). Measurements were always taken from the top of the leaf and the values for the five largest rosette leaves were averaged. Eight replicate plants were analysed per treatment. Wild type and the mutant plants were grown in the same tray under same growth conditions. Student’s paired t-test, with a two-tailed distribution was used to analyse data.

### Senescence assays and other morphological traits during development

The onset of whole plant senescence was defined as the day on which the number of green leaves started to decline [48, 49]. Leaves were classified as senescent when more than half of the leaf area was yellow; otherwise leaves were classified as green. 16 replicate plants were analysed. Both mutant and wild-type were randomly distributed in the same tray. Student’s paired t-test, with a two-tailed distribution was used to analyse data.

### Semiquantitative RT-PCR

RNA was extracted from leaves using the Qiagen RNeasy kit. After DNA digestion with TURBO DNase (Ambion), first-strand cDNA was synthesized using SuperScript III reverse transcriptase (Invitrogen). PCR was performed for 30- 35 cycles with gene-specific primers as follows for SGR1(At4g22920): SGR1-L, 5′-acaagttcccatctccatgc-3′; and SGR1-R, 5′-ggaaaatgtcgcttcacgtt-3′. For SAG12 (At5g45890): SAG12-L, 5′-tccttacaaaggcgaagacg-3′; and SAG12-R, 5′-tcattaaccgggacatcctc-3′. For PPase (At1g13320): PPase-L, 5′-taacgtggccaaaatgatgc-3′, and PPase-R, 5′-gttctccacaaccgcttggt-3′. PCR products were visualized on an agarose gel stained with ethidium bromide. Fragment sizes for SGR1 (141 bp), SAG12 (93 bp), and PPase (61 bp) were all of the expected size.

### Sample preparation, probe labelling and protein analysis

Probes were synthesised previously: DCG-04 [50], AMS101 [51] and MV151 [43]. Proteins were extracted from 100 mg of homogenised frozen leaves in 0.5 ml water for DCG-04 labeling or 100 μL water for labeling with other probes. Debris was removed by centrifugation (2 min at 16000 g). Labelling was conducted in 60 μl of protein extract containing 70 mM sodium acetate buffer (NaOAc) with probe-dependent pH, 1 mM DTT and 0.2 or 2 μM DCG-04, 2 μM AMS101 and 2 μM MV151. Extracts labelled with DCG-04 were incubated for 5 hours at room temperature (22–25 °C) with continuous mixing, while samples labelled with fluorescent probes AMS101 and MV151 were incubated for 2 hours at room temperature in the dark. Equal dilutions of DMSO were added to the no-probe controls. Preincubation with 1 mM E-64 added as a control for samples incubated with DCG-04 and MV151 for detection of PLCPs. The labelling reaction was stopped by adding 4x SDS-PAGE loading-buffer containing β-mercaptoethanol and then proteins were separated by 12% SDS PAGE. Labelled proteins were visualized by in-gel fluorescence scanning using a Typhoon 9000 scanner (GE Healthcare Life Science, http://www.gelifesciences.com) with excitation and emission at 532 and 580 nm respectively, or transferred to a membrane and analysed using streptavidin-HRP. Anti-RD21 and anti-AALP antibodies were described previously [25, 52].

### Affinity purification and identification of labelled proteins

Selected leaves of 6-week old plants growing in 12/12 hour light conditions were covered with aluminium foil for 7 days to induce senescence. Proteins were then extracted from five leaves into 2-4 mL water with subsequent centrifugation for 5 min at 20000 g. Supernatant was diluted with 1 M labelling buffer (1 M NaOAc, pH 6) to the final concentration of 50 mM and protein concentration of 5 mg/mL. The protein extract was labelled with 1 μM DCG-04 in the presence of 1 mM DTT for 2 hours at room temperature with gentle agitation. The labelled protein extract was applied to a PD10 column that had been equilibrated with 50 mM Tris-HCl, pH 8. Desalted samples were incubated with 100 μl avidin beads for 1 hour under gentle agitation. Avidin beads were collected by centrifugation for 5 min at 2000 rpm. Beads were washed twice with 1% SDS and twice with 6 M Urea, once with 50 mM Tris pH 8, once with 0.1% (w/v) Tween 20 and once with water. Beads were then incubated in 100 mM DTT for 20 min under gentle agitation, followed by incubation in 100 mM iodoacetamide under gentle agitation in the dark for 20 min. After washing three times in water, loading buffer was added to the beads and proteins separated by 12% SDS-PAGE. Labelled proteins in gels were visualized by Sypro Ruby staining. The visualized protein bands were excised and placed into 1.5 ml Eppendorf tubes. The slices were washed with 500 ml of 100 mM ammonium bicarbonate (Sigma) twice for 15 min. Proteins were reduced with Tris(2-carboxyethyl)-phospine (Sigma) for 30 min at 62 ° C and alkylated with 55 mM iodoacetamide for 30 min at room temperature. Gel fragments were washed three times for 15 min in 50:50 acetonitrile: 100 mM ammonium bicarbonate and dehydrated with 50 μl of 100% acetonitrile. Acetonitrile was removed and gel fragments were dried using an Eppendorf SpeedVac for 5 minutes. Gel slices were incubated in 25 mM ammonium bicarbonate and 10 ng μL^-1^ trypsin overnight at 37 °C. The supernatant was transferred to a new tube and gel slices were treated with 5% formic acid for 15 min at room temperature to inactivate trypsin. Gel slices were washed three times with 100% acetonitrile for 5 min. All supernatants were combined and concentrated in an Eppendorf SpeedVac to a final volume of approximately 10 μl. Tryptic peptides were analysed using a Thermo Scientific LTQ XL mass spectrometer according to [53].

## Results

### PLCPs and VPEs are amongst the major senescence-induced genes in leaves

To select proteases implicated in leaf senescence, we compared the transcript levels for Arabidopsis protease-encoding genes in green and senescent leaves from a recently published leaf development time course [54]. We binned these proteases into 41 protease families according to the MEROPS peptidase database [7]. On average, the highest transcript levels in senescent leaves (>1000 fragments per kilobase per million, FPKM) were observed for Aspartic proteases (clan AA, family A1), PLCPs (clan CA, family C1A), VPEs (clan CD, family C13), and Clp endopeptidases (clan SK, family S14) (Additional file 2: Figure S1A). We focused our attention to VPEs and PLCPs because those families contained the most senescence-induced protease genes, and because their average expression change was higher than 2 fold, and we have tools to monitor their activity.

The largest increase in transcript level in the PLCP C1A group was for *SAG12*, which showed a 1934-fold induction, dominating the PLCP transcript levels at 2145 ± 690 FPKM (Additional file 2: Figure S1B and Additional file 3: Table S1), consistent with *SAG12* being a major senescence-specific marker gene [18–20]. Besides *SAG12*, transcript levels in senescent leaves were also high and induced more than two-fold for *RD21A* (3.7-fold, 945 ± FPKM), *CTB3* (9.5-fold, 427 ± 108 FPKM), *RD19A* (2.1-fold, 814 ± 73 FPKM), *RD19C* (2.6-fold, 1038 ± 42 FPKM) and *AALP* (3.0-fold, 721 ± 96 FPKM) (Additional file 2: Figure S1B and Additional file 3: Table S1). However, at the overall mRNA level, transcripts of *RD21A, SAG12, RD19A, RD19C, AALP* and *CTB3* dominated the PLCP transcriptome of senescent leaves. Transcript levels of all *VPE* genes are upregulated during senescence but only levels of *γVPE* transcripts were relatively high (963 ± 100 FPKM) in the transcriptome of senescing leaves (Additional file 2: Figure S1B and Additional file 3: Table S1).

### Senescing leaves have increased PLCP and decreased VPE activities

**Fig. 1:**
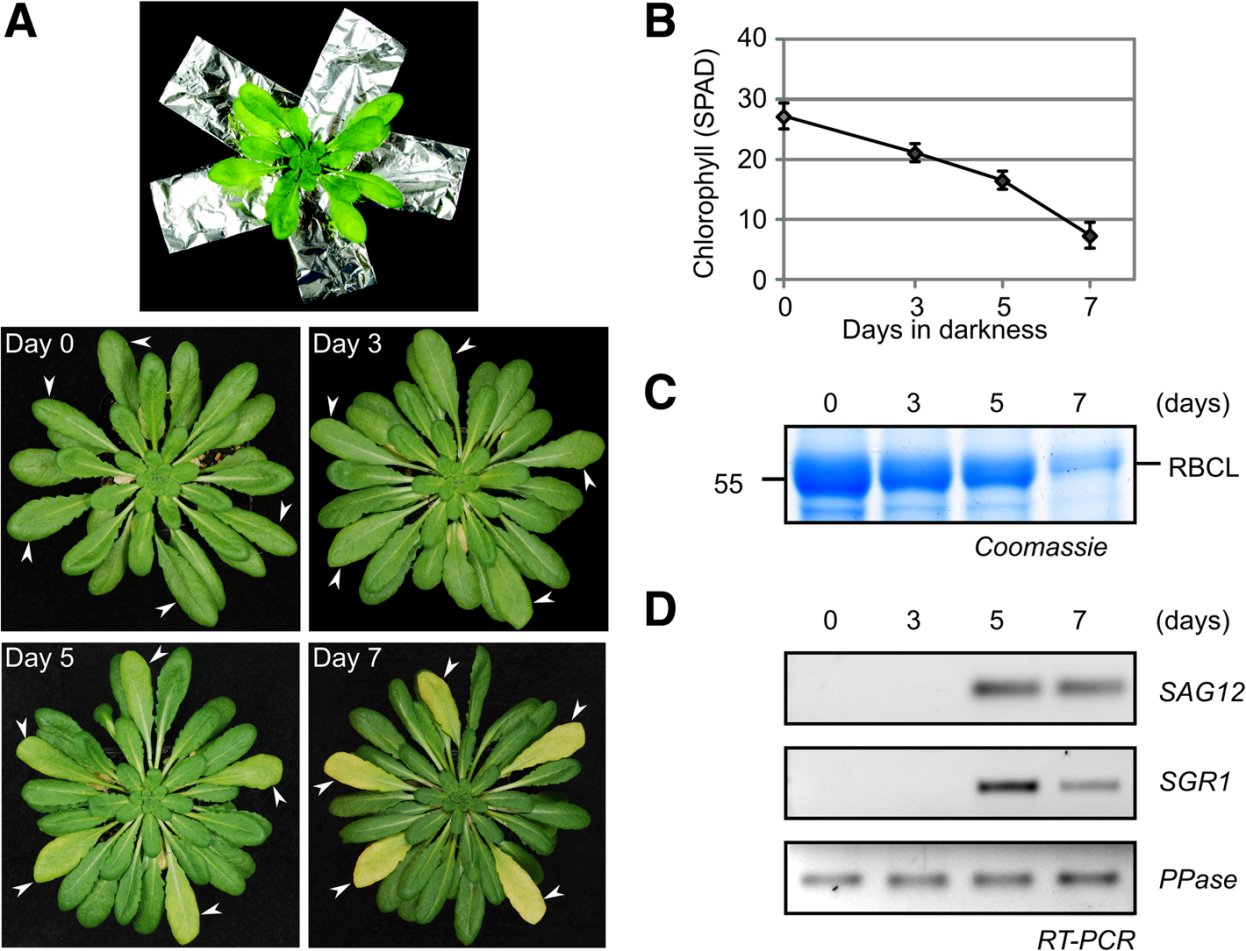
Individually darkened leaves on intact plants. **a** Five individually darkened leaves covered with aluminium foil. Arabidopsis plants (Col-0) were grown under short day conditions (8/16 hours day/night cycles). **b** Changes in chlorophyll ratio in individually darkened leaves. Each point represents the mean of means of 5 leaves from 4 individual plants with standard deviation (c) Changes in abundance of the large subunit of Rubisco (RBCL) in individually darkened leaves. **d** Expression of SAGs markers in individually darkened leaves: *SAG12*, *SGR1* (*Stay Green Gene 1*), and PPase (*Protein Phosphatase 2A Subunit A3,* control)

To induce senescence in Arabidopsis, we individually darkened leaves of approximately 8-week-old Arabidopsis plants (grown in 12/12 and 8/16 hours day/night light cycles, respectively) by covering leaves with aluminium foil for up to seven days [55] (Fig. 1a). Aluminium foil was lined inside with dark plastic and the leaves remained attached to the plant. The five to six largest leaves with approximately the same size and age were chosen for covering on each plant. This system resembles shading in nature and induces natural degradation of chlorophyll and the large subunit of Rubisco (Fig. 1b and c). Under these conditions, the expression of senescence marker gene *SAG12* and *SGR1* (*Stay Green Gene 1,* [56]) were induced (Fig. 1d), demonstrating that this treatment induces the classical senescence program. We used these individually darkened leaves for subsequent experiments.

**Fig. 2:**
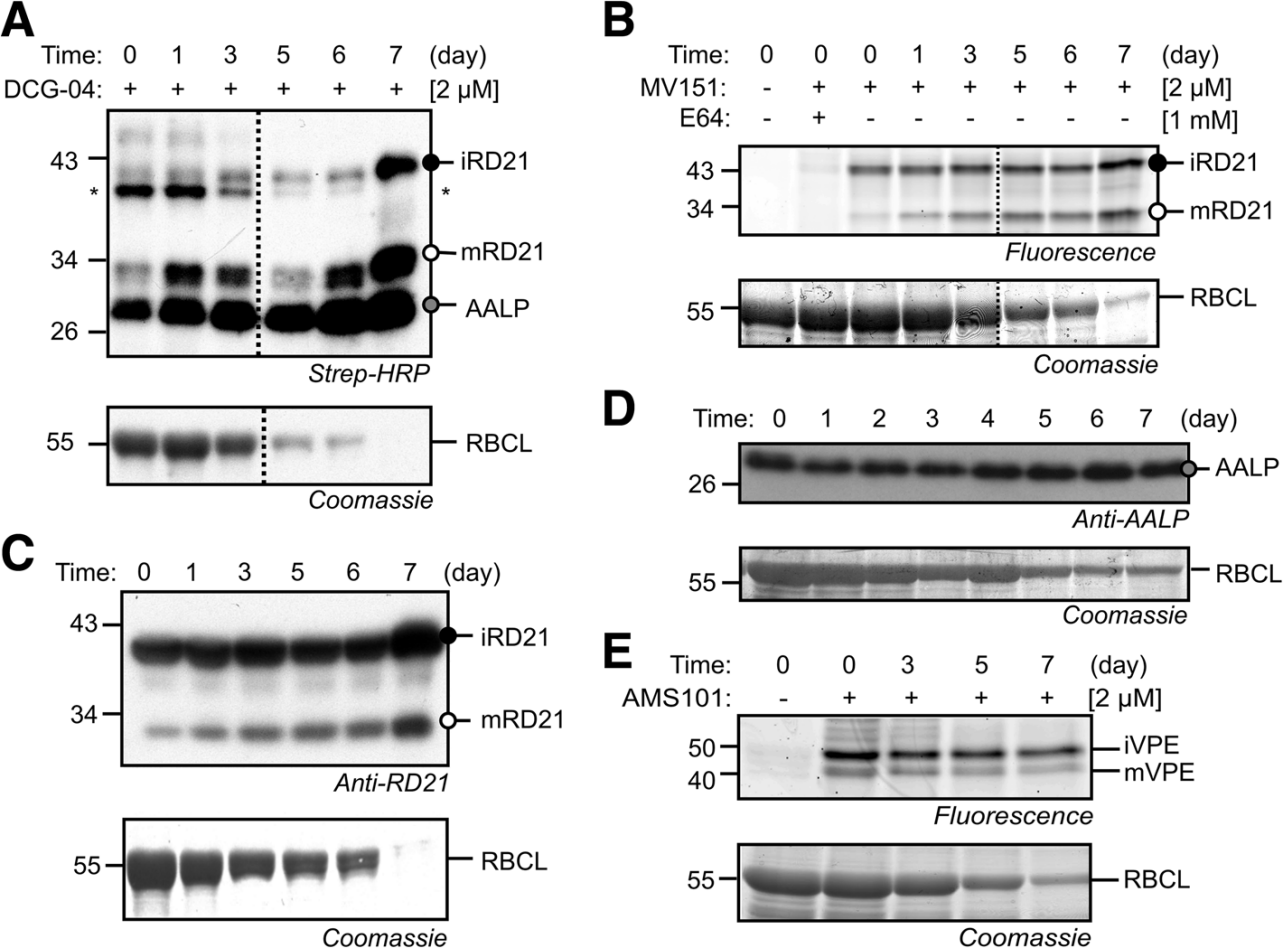
Induced PLCP and reduced VPE activities individually darkened leaves. Increased DCG-04 (**a**) and MV151 (**b**) labeling of PLCPs during senescence. Leaf extracts of equal fresh weights of individually darkened leaves were labeled for 5 hours with 2 μM DCG-04 at pH 6.5 or 2 μM MV151 at pH 4.5 and biotinylated proteins were detected using streptavidin-HRP (**a**) or fluorescent proteins were detected by scanning (**b**), respectively. *, endogenously biotinylated protein. **c, d** Accumulation of RD21A (**c**) and AALP (**d**) proteins in individually darkened leaves. Protein extracts of equal fresh weights of individually darkened leaves were separated and detected from protein blots using RD21A and AALP –specific antibodies, respectively. **e** Reduced AMS101 labeling of VPEs in individually darkened leaves. Leaf extracts of equal fresh weights of individually darkened leaves were labeled for 2 hours with 2 μM AMS101 at pH5.5 and analysed by fluorescence scanning. Coomassie stains of the membranes (**a-d**) or protein gel (**e**) is used as a control to show degradation of the large subunit of Rubisco (RBCL). The dotted line (**a, b**) indicates a removed lane from a western blot

To study the activity of PLCPs during leaf senescence by ABPP, we labeled leaf extracts of individually darkened leaves with DCG-04, a biotinylated chemical probe based on PLCP inhibitor E-64 [50, 57]. Detection of biotinylated proteins revealed increased intensities of signals migrating at 25, 30 and 40 kDa, which represent AALP, mature (m) and intermediate (i) RD21A, respectively (Fig. 2a, [43, 57]). Preincubation with an excess of E-64 prevented labeling of these proteins, suggesting that the signals represent PLCPs (Additional file 2: Figure S2). To confirm the increased PLCP labeling, we labeled the same proteomes with MV151, a fluorescent probe that can label a subset of the PLCPs, including RD21A [43]. MV151 labeling displayed increased intensities of 30 and 40 kDa signals, which are likely to represent mRD21A and iRD21A, respectively (Fig. 2b, [43]). To study accumulation of RD21A and AALP proteins, we performed western analysis using RD21A and AALP antibodies [25, 52]. Consistent with the increased labeling, we found that RD21A and AALP proteins also accumulate in individually darkened leaves, concomitantly with decreasing amounts of the large subunit of Rubisco (Fig. 2c and d).

To monitor the activity of VPEs in individually darkened leaves we labeled leaf extracts with AMS101, a fluorescent activity-based probe for VPEs [51]. AMS101 detected signals at 40 and 43 kDa, which likely represent immature and mature isoforms of γVPE because this causes the major VPE activity in green leaves (Fig. 2e, [51]), and *γVPE* transcript level dramatically increases in senescent leaves (Additional file 2: Figure S1B). However, the intensity of this signal decreased during senescence (Fig. 2e), despite upregulated *VPE* transcript levels.

### Many PLCPs have increased activity in senescing leaves

**Fig. 3:**
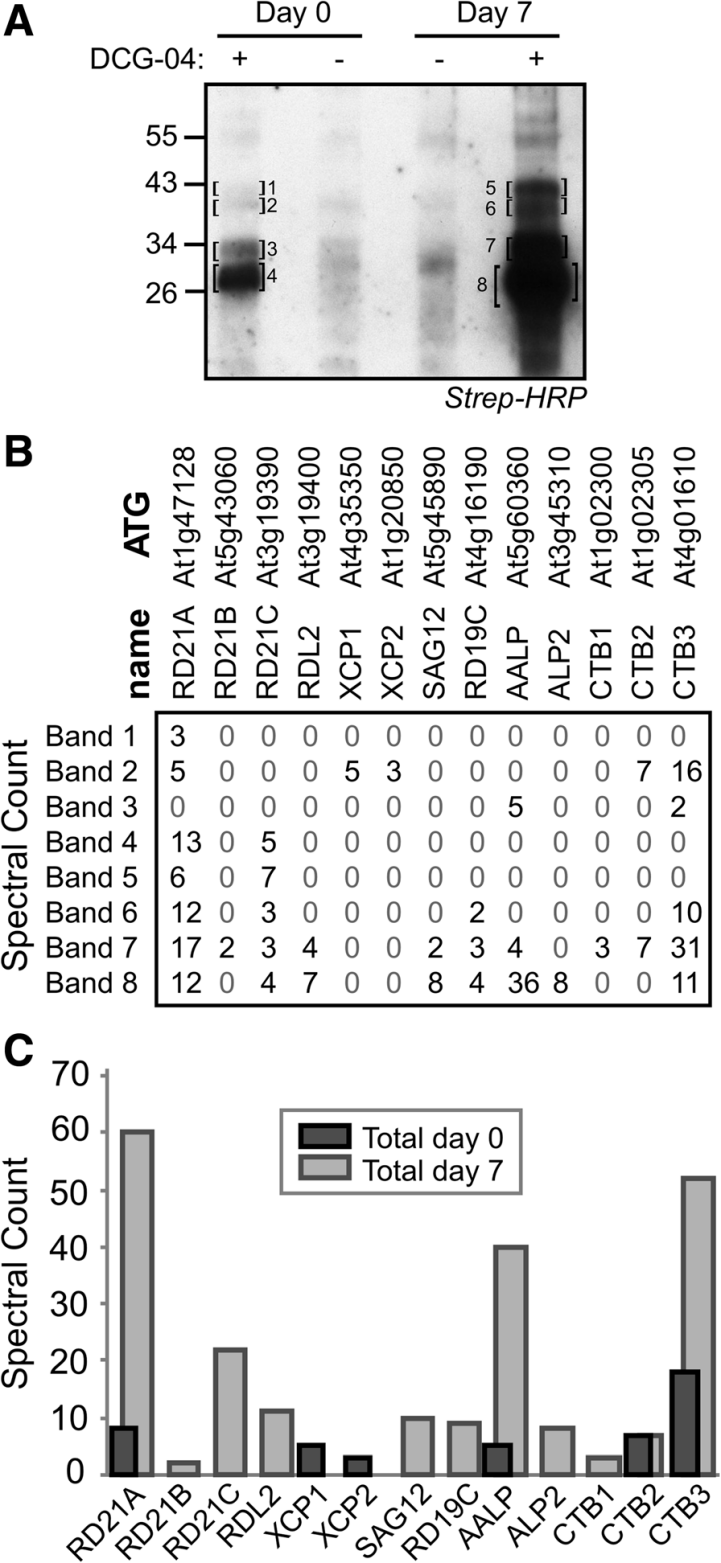
Extracts of senescent leaves contain more active PLCPs. **a** Profile of purified DCG-04-labelled and un-labelled proteins of control (day 0) and senescent leaves (day 7). Biotinylated proteins were purified from DCG-04 labelled proteomes using avidin beads. Four gel bands from control green leaves and four from yellow senescent leaves were excised and treated with trypsin. Eluted peptides were analysed and identified by MS/MS. **b** Spectral counts for identified PLCPs in the eight individual bands. **c** Sum of the total spectral counts over the 13 identified proteases, divided over green (day 0) and senescent (day 7) leaves

To identify the proteins labelled with DCG-04 extracted from individually darkened senescent leaves, leaf extracts generated at days 0 and 7 were labeled with and without DCG-04 and biotinylated proteins were purified and separated on protein gels. Biotinylated proteins increased dramatically in abundance at day 7 when compared to day-0 control leaves, and most signals were absent in the no-probe-controls (Fig. 3a). Eight protein band regions were excised, treated with trypsin, and analysed by mass spectrometry. Peptides from eleven PLCPs were detected, including SAG12 (Fig. 3b). The highest number of spectral counts in senescent leaves were from RD21A, followed by CTB3, AALP, RD21C, RDL2, SAG12, RD19C, ALP2, CTB2, CTB1 and RD21B (Fig. 3c). Notably, peptides from XCP1 and XCP2 were detected only in green leaves and were not identified in senescent leaves (Fig. 3c), consistent with reduced transcript levels (Additional file 2: Figure S1B). All other detected proteases seem to have higher activity levels in senescing leaves (Fig. 3c).

### RD21A and AALP are the dominant active PLCPs in senescing leaves

**Fig. 4:**
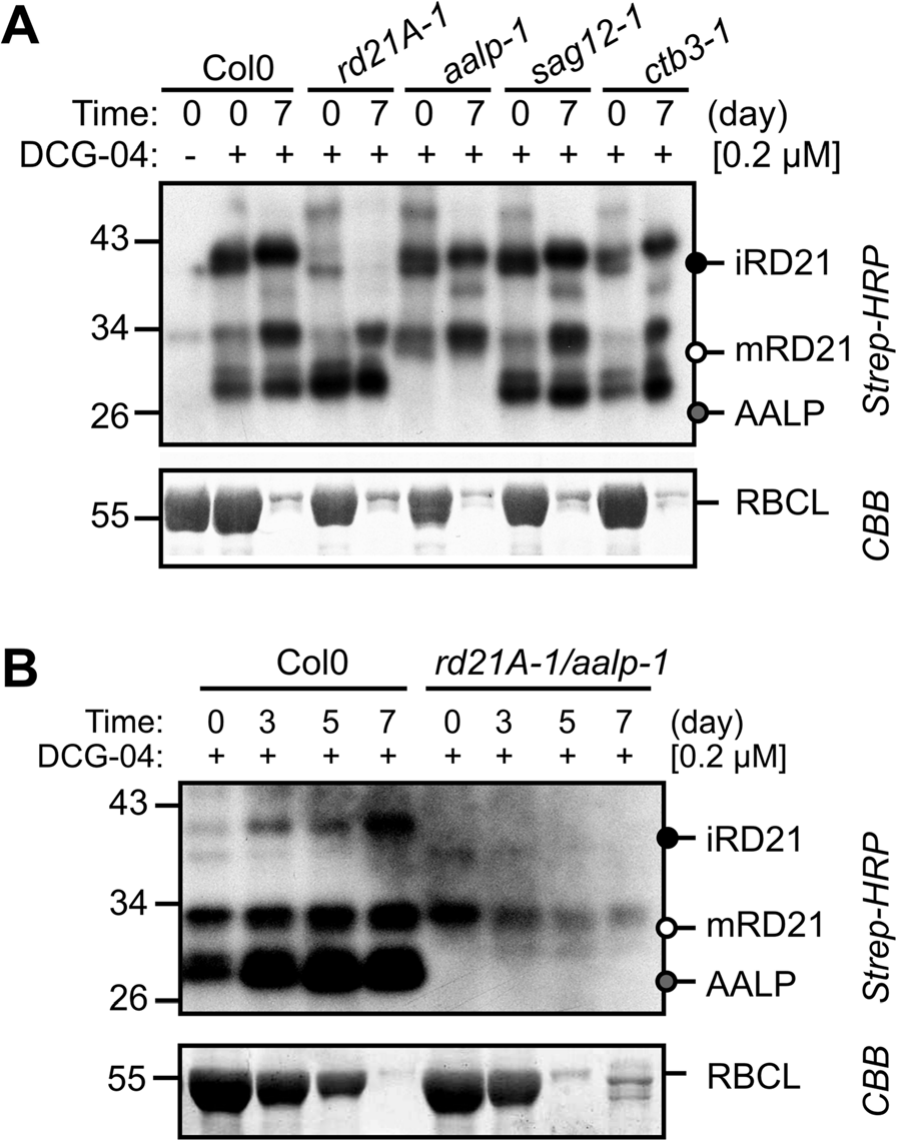
Major PLCP activities are depleted in senescent leaves of *rd21A-1/aalp-1* double mutant plants without affecting Rubisco levels. **a** PLCP activity profiles of control (day 0) and senescent leaf (day 7) of *rd21A-1*, *aalp-1*, *sag12-1* and *ctb3-1* mutants and wild-type plants. **b** PLCP activity profiles of individually darkened leaves at 0, 3, 5 and 7 days of the *rd21A-1/aalp-1* double mutant in comparison to wild-type (Col0) plants. Leaf extracts of equal fresh weights of individually darkened leaves were labeled for 5 hours with 0.2 μM DCG-04 at pH 6.5 and biotinylated proteins were detected from protein blots using streptavidin-HRP

To confirm the identity of the proteases causing the major signals in the DCG-04 activity profile of senescent leaves, we labeled leaf extracts of green and senescent leaves with DCG-04 of the *sag12-1*, *rd21A-1*, *ctb3-1* and *aalp-1* null mutants [42]. We only detected an altered protease activity profile for *rd21A-1* and *aalp-1* mutants (Fig. 4a). The 40 signal was absent and the 30 kDa signal strongly reduced in the *rd21A-1* mutant and the 25 kDa signal was missing in the *aalp-1* mutant, indicating that these signals are caused by RD21A and AALP, respectively. Consistently, the *rd21A-1/aalp-1* double mutant lacks all three major signals (Fig. 4b), indicating that RD21A and AALP are the major PLCP activities in senescing leaves, and that no other PLCP compensates for the reduced PLCP activity in this double mutant. Importantly, despite the absence of major PLCP activities in these plants, the decline of rubisco levels is not reduced in the *rd21A-1/aalp-1* mutant plants (Fig. 4b, bottom).

### PLCP and VPE protease mutants do not have a strong senescence phenotype

To study the contribution of PLCPs and VPEs to senescence in individually darkened leaves further, we subjected the following PLCP mutant lines to senescence assays: *rd21A-1*, *aalp-1*, *sag12-1*, *ctb3-1*, *rd21A-1/aalp-1* double mutant, and *ctb1/ctb2/ctb3* triple mutant [42, 46]. We also included the RD21A overexpressor under the control of the 35S promoter (35S::RD21A, Additional file 2: Figure S3). In addition, we included the quadruple VPE null mutant (*qvpe*) which lacks all four VPEs [45] and an overexpressor of γVPE (35S::γVPE, [44]) which might prevent the γVPE decline during senescence.

**Fig. 5:**
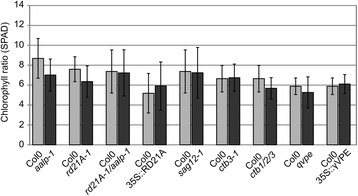
Chlorophyll ratio is unaltered in individually darkened leaves for Cys protease mutant- and overexpressor lines**.** Chlorophyll ratio was measured with a SPAD meter on at least six leaves covered with aluminium foil for seven days from a total of eight plants. Error bars represent standard deviation of n = 8 biological replicates

**Fig. 6:**
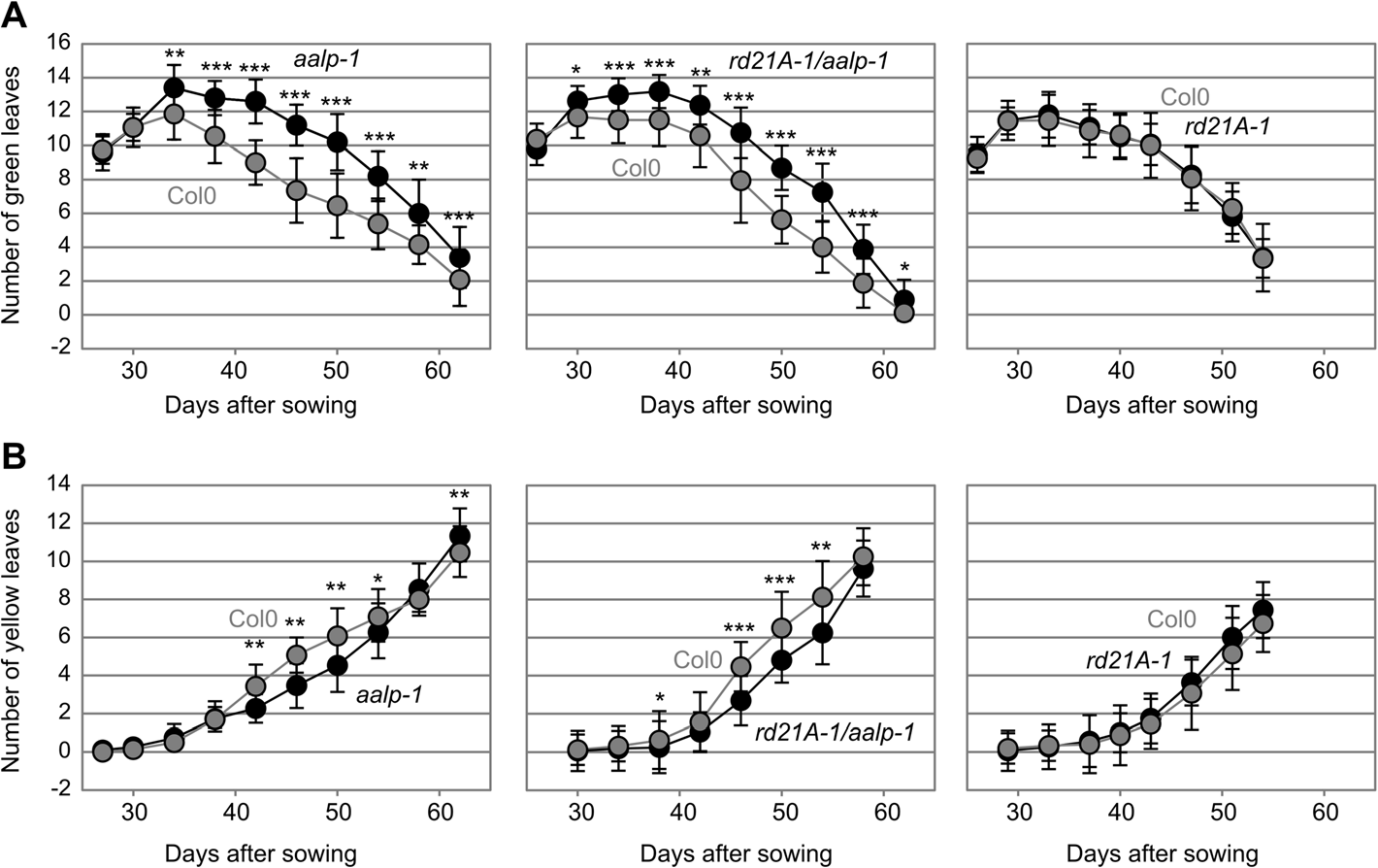
Delayed onset of whole plant senescence in *aalp-1* and *rd21A-1/aalp-1* mutants. Plants were grown under long day conditions (16/8 day/night). Number of green (**a**) and yellow (/senescent) (**b**) leaves at different time points after the sowing of wild-type plants and *aalp-1*, *rd21A-1/aalp-1* and *rd21A-1* mutants. Each data point represents the mean of 16 plants with standard deviation from one representative experiment. The experiments were repeated twice with similar results

We measured the chlorophyll ratio in leaves individually darkened for seven days after the plants were grown under short day conditions for eight weeks. The data did not show any significant difference between tested lines and their wild-type control (Fig. 5). However, this senescence assay displays senescence of individual leaves but not the whole plant. We therefore expanded our senescence assays to plants grown under long day conditions (16/8 hours day/night cycles) to study natural senescence of rosette leaves induced upon flowering. We monitored the number of green and senescent leaves at different time points during development under long day conditions. Interestingly, the *aalp-1* and *rd21A-1/aalp-1* mutants showed significantly more green leaves and less senescent leaves at early stages of developmental senescence than wild-type plants (Fig. 6a and b). By contrast, other mutants (*rd21A-1, sag12-1, ctb3-1, ctb1/2/3* and *qvpe* mutants) and lines overexpressing RD21A or γVPE (35S::RD21A and 35S::γVPE ) did not show significant differences in the number of green and senescent leaves (Additional file 2: Figure S4). The fact that both *aalp-1* and *rd21A-1/aalp-1* mutants, but not the *rd21A-1* mutant, showed this whole plant senescence phenotype indicates that the *aalp-1* mutation correlates with this delayed progression of the senescence phenotype. We were unable to identify independent *aalp-1* null mutant alleles for verification of this phenotype. Thus at this stage we cannot exclude that the senescence phenotype is caused by the absence of AALP or originates from a secondary, unidentified mutation that co-segregated into the *rd21A-1/aalp-1* double mutant.

## Discussion

In this study we showed that, while *PLCP* and *γVPE* -encoding genes are induced transcriptionally during senescence, ABPP probes showed that only PLCPs had increased activity in individually darkened leaves of Arabidopsis. Yamada *et al.* [25] previously showed that RD21A protein levels increase during developmental senescence of Arabidopsis. Increasing activities of PLCPs using the DCG-04 probe have also previously been observed during developmental leaf senescence in Arabidopsis and in wheat leaf-segments incubated in the dark [57, 58]. However the identity of these active proteases in senescent Arabidopsis leaves was not previously known. In this work, 11 active proteases were purified and identified from senescent leaves: RD21A, CTB3, AALP, RD21C, RDL2, SAG12, RD19C, ALP2, CTB2, CTB1 and RD21B. Four detected PLCPs (SAG12, RD19C, CTB2 and CTB1) have not previously been detected by DCG-04 labelling in green leaves or other Arabidopsis organs [57, 59]. Our data demonstrate that these proteases are active in extracts of senescent leaves.

We have analysed mutants for senescence-associated PLCPs and *γ*VPE proteases for senescence phenotypes, as chlorophyll content in individually darkened leaves and the number of green and senescent leaves in naturally senescing plants. Surprisingly, none of the mutant lines showed any phenotype in individually darkened leaves, despite the evident lack of major protease activities displayed by DCG-04 labeling in these lines. The following lines did also not show any obvious phenotype during developmental senescence: *rd21A-1*, *sag12-1*, *ctb3-1*, *ctb1/2/3, qvpe, 35S::RD21A* and *35S::γVPE*, consistent with previously published results for *sag12* and *ctb3* mutants [2, 45].

The fact that single PLCP and VPE mutants showed no senescence phenotype may be due to redundancy between proteases. For instance, Arabidopsis *CTB* genes were reported to act redundantly in leaf senescence because only the triple *ctb1/2/3* mutant showed delayed senescence in detached leaves incubated in the dark [45]. In our senescence assay, however, the *ctb1/2/3* mutant has no senescence phenotype, possibly because different senescence assays induce different regulatory pathways [60]. For instance the 50 kDa Rubisco cleavage fragment is present only in detached leaves and under low light but not in leaf segments exposed to high light and in intact plants induced to senesce by N-deprivation [16]. Leaf senescence is also associated with the loss of water, so it is possible that the drought-responsive *RD19A* and *RD21A* genes [61] are upregulated at the late stages of dark-induced senescence due to the loss of the water, not because they are involved in the senescence process itself.

The delayed progression of senescence in *aalp-1* and *rd21A-1/aalp-1* mutants suggests that AALP contributes to the senescence process. Our observation is consistent with the report that suppression of the *AALP* orthologue in Broccoli delays senescence in florets [62]. AALP is a predicted aminopeptidase similar to mammalian cathepsin-H. This protease cannot act as an endopeptidase because one side of the substrate binding groove is blocked by a minipeptide that originates from the prodomain and remains covalently bound through a disulphide bridge [63]. AALP shares these features and therefore probably acts on (neo) N-termini during the bulk protein degradation process in the vacuole. The delay in senescence in *aalp-1* null mutants may be the result of an imbalance in amino acid availability.

## Conclusions

Senescing Arabidopsis leaves show a massive transcriptional activation encoding Cys proteases, especially vacuolar processing enzymes (VPEs) and papain-like Cys proteases (PLCPs). Protease activity profiling demonstrates that in contrast to increased VPE transcript levels, VPE activity is not induced. By contrast, senescing leaves have an increased activity of PLCPs, and MS and mutant analysis show that this increased PLCP activity is dominated by RD21 and AALP. VPE and PLCP mutant and overexpressor lines do not show an altered rubisco degradation or chlorophyll ratio phenotypes. In whole plant senescence assays, however, *aalp-1* and *aalp-1/rd21-1* mutants show a delayed senescence, suggesting a role for AALP in developmental senescence. Taken together, these data indicate that Cys proteases play redundant roles in leaf senescence.

## Additional files

Additional file 1: Table S2. Arabidopsis knock-out and over-expressor lines used in this study. (DOC 32 kb)
Additional file 2: Figure S1. Transcript levels of protease-encoding genes in mature and senescing leaves of Arabidopsis. (**A**) Transcript levels in FPKM of genes grouped per protease family. Transcript levels in FPKM were extracted from GSE43616 (Woo et al., 2016) and were summed up for each protease according to the protease families of the MEROPs database. (**B**) Transcript levels of genes encoding PLCPs (top) and VPEs (bottom). Shown are the transcript levels in mature green leafs (left, 16D+18D), senescent leaves (middle, 28D+30D) and the ratio between green and senescence leaves (right). Error bars represent standard deviation (STDEV) of (n=4) samples. **Figure S2.** E-64 suppresses DCG-04 labeling mature and senescent leaves. Leaf extracts of equal fresh weights of individually darkened leaves were preincubated with 2 μM E-64 for 30 minutes and then labeled for 5 hours with 2 μM DCG-04 at pH 6.5 and biotinylated proteins were detected using streptavidin-HRP or fluorescence scanning, respectively. *, endogenously biotinylated proteins. **Figure S3.** Characterization of the transgenic 35S::RD21 Arabidopsis line. The homozygous progeny of a Col-0 plant transformed with pRH628 carrying 35S::RD21 is compared to the wild-type (Col-0) and to two *RD21* knock-out mutants: *rd21-1* and *rd21-2*. Leaf extracts were labeled with DCG-04 and biotinylated proteins were detected on protein blots using streptavidin-HRP. **Figure S4.** No altered natural senescence in other PLCP/VPE mutants and overexpressor lines. Number of green leaves at different time points of wild-type and mutant plants grown at long days. Error bars represent STDEV of n=16 biological replicates. (PDF 469 kb)
Additional file 3: Table S1. Transcript levels of proteases in non-senescent and senescent leaves. (XLSX 24 kb)

## Abbreviations

AALP: Arabidopsis aleurain-like protease, ALP, aleurain-like protease
ABPP: Activity-based protein profiling
CTB: Cathepsin B
PAO: Pheophorbide-a-oxygenase
PLCP: Papain-like Cys protease
RD19: Responsive-to-desiccation 19
RD21: Responsive-to-desiccation 21
RDL: RD21-like protease
SAG12: Senescence-associated gene 12
SGR1: Stay Green Gene 1
VPE: Vacuolar processing enzyme
XCP: Xylem-specific protease
