# Supplementary material for: Major Cys protease activities are not essential for senescence in individually darkened Arabidopsis leaves
Source: BMC Plant Biol. 2017 Jan 6;17:4. doi: 10.1186/s12870-016-0955-5 (PMC5217659; doi:10.1186/s12870-016-0955-5)
Supplement: Additional file 1: Table S2. — Arabidopsis knock-out and over-expressor lines used in this study. (DOC 32 kb) [file 12870_2016_955_MOESM1_ESM.doc]

**Table S2:** Arabidopsis knock-out and over-expressor lines used in this study

| **Mutant** | **Bckgrnd** | **Gene** | **Line** | **Reference** |
| --- | --- | --- | --- | --- |
| *rd21-1* | Col-0 | At1g47128 | SALK_090550 | Wang et al., 2008 |
| *aalp-1* | Col-0 | At4g60360 | SALK_075550 | Wang et al., 2008 |
| *sag12-1* | Col-0 | At5g45890 | SALK_124030 | Wang et al., 2008 |
| *ctb3-1* | Col-0 | At4g01610 | SALK_019630 | Wang et al., 2008 |
| *rd21-1/aalp-1* | Col-0 | At1g47128  At4g60360 | SALK_090550  SALK_075550 | Gu et al., 2012 |
| *ctb1/2/3*  *(line #64-4)* | Col-0 | At1g02300  At1g02305  At4g01610 | SALK_151526  RNAi  SALK_019630 | McLellan et al., 2009 |
| *qvpe* | Col-0 | At2g25940  At1g62710  At4g32940  At3g20210 | αvpe::dSpm1  βvpe::dSpm1  SALK_010372 δvpe::dSpm1 | Gruis et al., 2004 |
| *35S::γVPE* | Col-0 | At4g32940 | OE | Rojo et al., 2003 |
| *35S::RD21A* | Col-0 | At1g47128 | OE | This manuscript |
