# Supplementary material for: Major Cys protease activities are not essential for senescence in individually darkened Arabidopsis leaves
Source: BMC Plant Biol. 2017 Jan 6;17:4. doi: 10.1186/s12870-016-0955-5 (PMC5217659; doi:10.1186/s12870-016-0955-5)
Supplement: Additional file 2: Figure S1. — Transcript levels of protease-encoding genes in mature and senescing leaves of Arabidopsis. (A) Transcript levels in FPKM of genes grouped per protease family. Transcript levels in FPKM were extracted from GSE43616 (Woo et al., 2016) and were summed up for each protease according to the protease families of the MEROPs database. (B) Transcript levels of genes encoding PLCPs (top) and VPEs (bottom). Shown are the transcript levels in mature green leafs (left, 16D+18D), senescent leaves (middle, 28D+30D) and the ratio between green and senescence leaves (right). Error bars represent standard deviation (STDEV) of (n=4) samples. Figure S2. E-64 suppresses DCG-04 labeling mature and senescent leaves. Leaf extracts of equal fresh weights of individually darkened leaves were preincubated with 2 μM E-64 for 30 minutes and then labeled for 5 hours with 2 μM DCG-04 at pH 6.5 and biotinylated proteins were detected using streptavidin-HRP or fluorescence scanning, respectively. *, endogenously biotinylated proteins. Figure S3. Characterization of the transgenic 35S::RD21 Arabidopsis line. The homozygous progeny of a Col-0 plant transformed with pRH628 carrying 35S::RD21 is compared to the wild-type (Col-0) and to two RD21 knock-out mutants: rd21-1 and rd21-2. Leaf extracts were labeled with DCG-04 and biotinylated proteins were detected on protein blots using streptavidin-HRP. Figure S4. No altered natural senescence in other PLCP/VPE mutants and overexpressor lines. Number of green leaves at different time points of wild-type and mutant plants grown at long days. Error bars represent STDEV of n=16 biological replicates. (PDF 469 kb) [file 12870_2016_955_MOESM2_ESM.pdf]

SUPPLEMENTAL FIGURES Pružinská et al. Major Cys protease activities are not essential for senescence in individually darkened Arabidopsis leaves

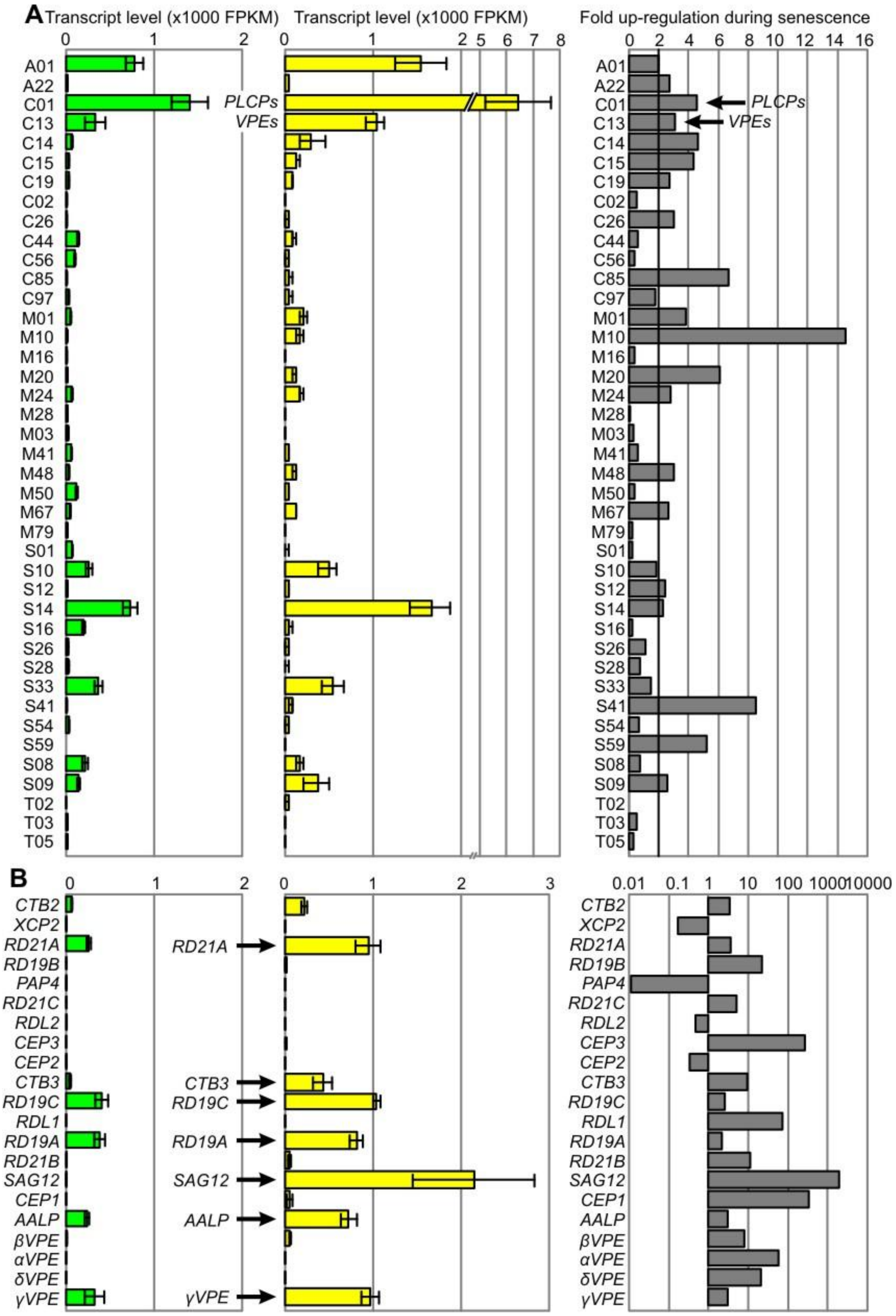

Figure S1. Transcript levels of protease-encoding genes in mature and senescing leaves of Arabidopsis. **(A)** Transcript levels in FPKM of genes grouped per protease family. Transcript levels in FPKM were extracted from GSE43616 (Woo et al., 2016) and were summed up for each protease according to the protease families of the MEROPs database. **(B)** Transcript levels of genes encoding PLCPs (top) and VPEs (bottom). Shown are the transcript levels in mature green leafs (left, 16D+18D), senescent leaves (middle, 28D+30D) and the ratio between green and senescence leaves (right). Error bars represent standard deviation (STDEV) of (n=4) samples.

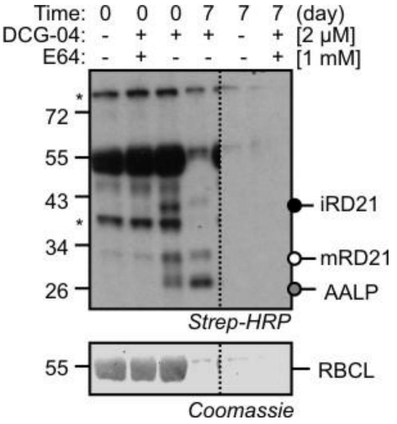

Figure S2. E-64 suppresses DCG-04 labeling mature and senescent leaves.

Leaf extracts of equal fresh weights of individually darkened leaves were preincubated with 2 µM E-64 for 30 minutes and then labeled for 5 hours with 2 µM DCG-04 at pH 6.5 and biotinylated proteins were detected using streptavidin-HRP or fluorescence scanning, respectively. *, endogenously biotinylated proteins.

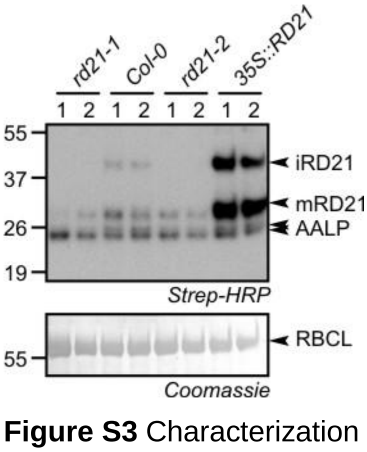

Figure S3 Characterization of the transgenic 35S::RD21 Arabidopsis line.

The homozygous progeny of a Col-0 plant transformed with pRH628 carrying 35S::RD21 is compared to the wild-type (Col-0) and to two *RD21* knock-out mutants: *rd21-1* and *rd21-2*. Leaf extracts were labeled with DCG-04 and biotinylated proteins were detected on protein blots using streptavidin-HRP.

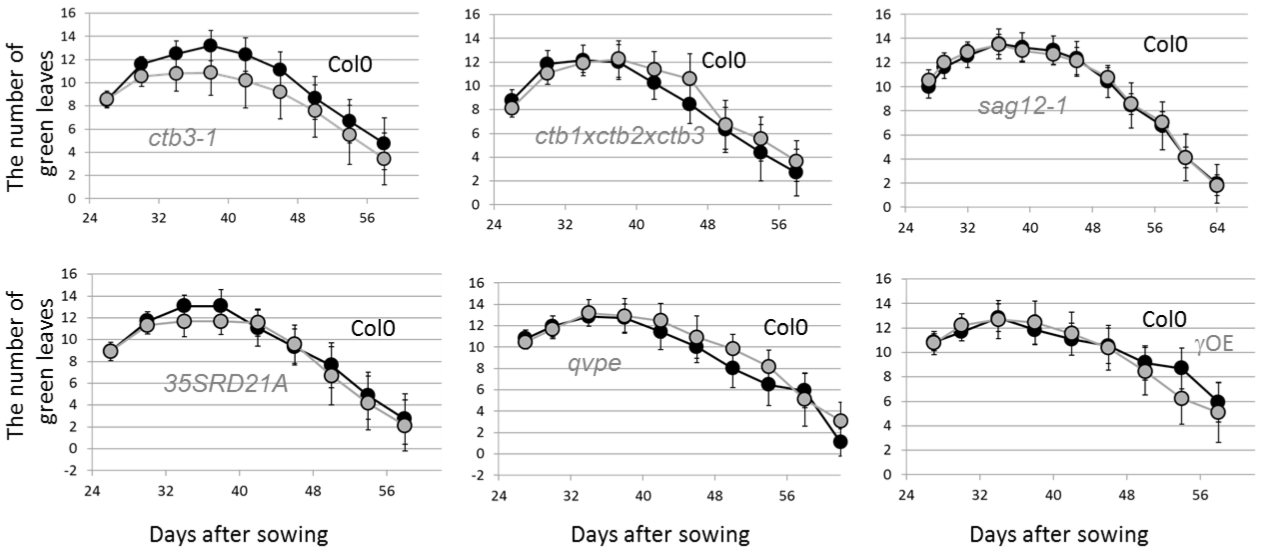

Figure S4: No altered natural senescence in other PLCP/VPE mutants and overexpressor lines. Number of green leaves at different time points of wild-type and mutant plants grown at long days. Error bars represent STDEV of n=16 biological replicates.
